# Poly[(μ_4_-3-carb­oxy­pyrazine-2-carboxyl­ato)(μ_4_-nitrato)dilithium]

**DOI:** 10.1107/S1600536812050738

**Published:** 2012-12-19

**Authors:** Wojciech Starosta, Janusz Leciejewicz

**Affiliations:** a Institute of Nuclear Chemistry and Technology, ul. Dorodna 16, 03-195 Warszawa, Poland

## Abstract

In the title compound, [Li_2_(C_6_H_3_N_2_O_4_)_2_(NO_3_)]_*n*_, the two symmetry-independent Li^I^ ions are each in a trigonal–bipyramidal coordination and are bridged by *N*,*O*-bonding ligands, forming mol­ecular ribbons propagating in [010]. Each Li^I^ ion is also coordinated by two O atoms from nitrate ions, connecting the ribbons into a three-dimensional network. Very strong intra­molecular O—H⋯O hydrogen bonds occur between the carboxyl and the carboxylate group.

## Related literature

For three structures of lithium(I) complexes with pyrazine-2,3-dicarboxyl­ate and water ligands, see: Tombul *et al.* (2008 ▶); Tombul & Güven (2009) ▶; Starosta & Leciejewicz (2011 ▶). For structures of calcium(II) complexes with the title ligand, see: Ptasiewicz-Bąk & Leciejewicz (1997 ▶); Starosta & Leciejewicz (2004 ▶, 2005*a* ▶,*b* ▶). 
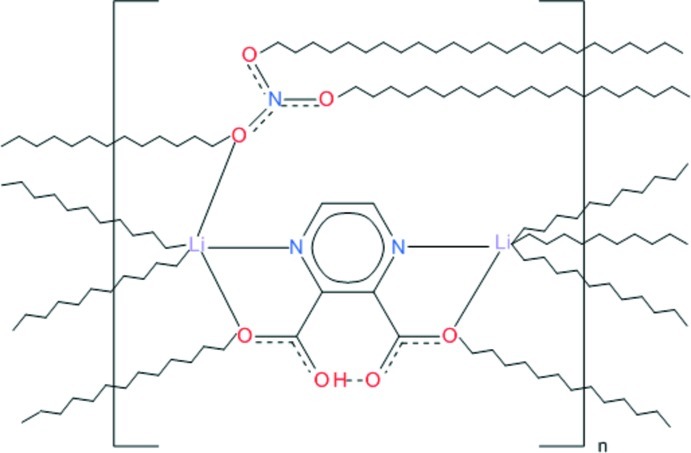


## Experimental

### 

#### Crystal data

[Li_2_(C_6_H_3_N_2_O_4_)_2_(NO_3_)]
*M* *_r_* = 241.99
Monoclinic, 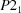

*a* = 4.6273 (1) Å
*b* = 15.8565 (3) Å
*c* = 6.1719 (2) Å
β = 95.598 (2)°
*V* = 450.69 (2) Å^3^
*Z* = 2
Mo *K*α radiation
μ = 0.16 mm^−1^
*T* = 293 K
0.20 × 0.14 × 0.12 mm

#### Data collection

Agilent SuperNova (Dual, Cu at zero, Eos) diffractometer
Absorption correction: multi-scan (*CrysAlis PRO*; Agilent, 2011 ▶) *T*_min_ = 0.936, *T*_max_ = 1.000
4032 measured reflections
2572 independent reflections
2401 reflections with *I* > 2σ(*I*)
*R*_int_ = 0.015

#### Refinement

*R*[*F* ^2^ > 2σ(*F* ^2^)] = 0.036
*wR*(*F* ^2^) = 0.084
*S* = 1.10
2572 reflections
167 parameters
1 restraint
H atoms treated by a mixture of independent and constrained refinement
Δρ_max_ = 0.21 e Å^−3^
Δρ_min_ = −0.20 e Å^−3^

### 

Data collection: *CrysAlis PRO* (Agilent, 2011 ▶); cell refinement: *CrysAlis PRO*; data reduction: *CrysAlis PRO*; program(s) used to solve structure: *SHELXS97* (Sheldrick, 2008 ▶); program(s) used to refine structure: *SHELXL97* (Sheldrick, 2008 ▶); molecular graphics: *SHELXTL* (Sheldrick, 2008 ▶); software used to prepare material for publication: *SHELXTL*.

## Supplementary Material

Crystal structure: contains datablock(s) I, global. DOI: 10.1107/S1600536812050738/kp2442sup1.cif

Structure factors: contains datablock(s) I. DOI: 10.1107/S1600536812050738/kp2442Isup2.hkl

Additional supplementary materials:  crystallographic information; 3D view; checkCIF report

## Figures and Tables

**Table 1:** Selected bond lengths (Å)

Li1—O1	2.086 (3)
Li1—O5	2.005 (3)
Li1—N1	2.158 (3)
Li1—O7^i^	1.994 (3)
Li1—O3^ii^	1.999 (3)
Li2—N4	2.176 (3)
Li2—O1^iii^	1.989 (3)
Li2—O5^iv^	2.014 (3)
Li2—O6^v^	2.040 (4)
Li2—O3	2.086 (3)

Symmetry codes: (i) 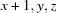; (ii) 
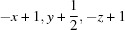
; (iii) 
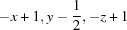
; (iv) 
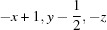
; (v) 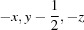.

**Table 2:** Hydrogen-bond geometry (Å, °)

*D*—H⋯*A*	*D*—H	H⋯*A*	*D*⋯*A*	*D*—H⋯*A*
O2—H1⋯O4	1.07 (4)	1.34 (4)	2.3955 (19)	170 (4)

**Fig. 1.**
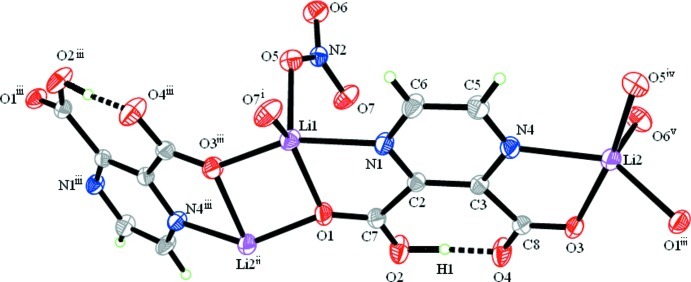
A fragment of the structure of the title compound with atom labelling scheme and 50% probability displacement ellipsoids. Symmetry code: (i) x + 1, y, z; (ii) -x + 1, y + 1/2, -z + 2; (iii) -x + 1, y - 1/2, -z + 2; (iv) -x + 1, y - 1/2, -z + 1; (v) -x, y - 1/2, -z + 1.

**Fig. 2.**
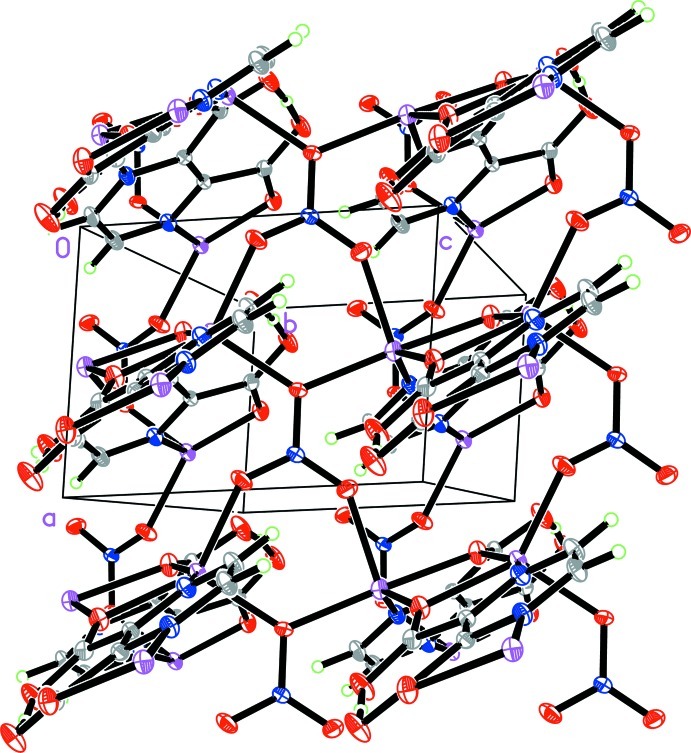
The packing of molecular ribbons in the structure of the title compound showing nitrate bridging mode.

## supplementary crystallographic information

### Comment

Pyrazine-2,3-dicarboxylate dianion shows large versality in forming coordination compounds with metal ions. Depending on the adopted chemical synthesis procedures, compounds with a number of different polymeric structures have been observed, as for example, in the case of the Ca(II) ion (Ptasiewicz-Bąk & Leciejewicz, 1997; Starosta & Leciejewicz, 2004, 2005*a*, 2005*b*). Polymeric structures of three Li^I^ complexes with the title ligand have been reported (Tombul *et al.*, 2008; Tombul & Güven, 2009; Starosta & Leciejewicz, 2011). Recently we have obtained a new compund with the title ligand. The asymmetric unit of the title compound contains two symmetry independent Li^I^ ions. Each shows a distorted trigonal-bipyramidal coordination geometry. The Li1 ion is coordinated by ligand *N1,O1* bonding group, a carboxylato O3^ii^ atom from the adjacent ligand and O5 and O7^i^ atoms from two different nitrate ions. The O1, O5, O7^i^ atoms form a base, the Li1 ion is 0.1572 (3) Å out of this plane; N1 and O3^ii^ atoms are at the axial positions. The same coordination geometry shows the Li2 ion which is situated 0.3616 (3) Å out of the equatorial plane composed of N4, O1^iii^ and O6^v^ atoms, while the O3 and O5^iv^ atoms form the apices. The observed Li—O and Li—N bond distances are typical of Li^I^ complexes with diazine carboxylate ligands. Ligand carboxylate O2 and O4 atoms remain coordination inactive. Fourier maps indicate clearly, that the O2 atom is protonated acting as a donor in a low-barrier intramolecular hydrogen bond of 2.3955 (19) Å to the O4 atom suggesting a partial proton transfer(Table 2). The ligand is monovalent and with the nitrate anion maintains the charge balance in the structure. Pyrazine ring is planar with r.m.s. of 0.0051 (2) Å; carboxylate groups C7/O1/O2 and C8/O3/O4 form with it dihedral angles of 8.4 (1)° and 12.5 (1)°, respectively. Ligand molecule bridges metal ions in µ_4_ mode. Li1 and Li2 ions are chelated by both *N,O* groups of a ligand and bidentate O1^ii^ and O3^ii^atoms [Fig. 1]. A dimeric moiety Li1/O1/L2^ii^/O3^iii^ constitutes a link in a bridging pathway formed by ligand molecules, giving rise to molecular ribbons propagating in the [010] direction. A nitrate anion with r.m.s. of 0.0016 (1) Å acts also in the µ_4_ mode and forms the other bridging pathway: while the O6 atom coordinates the Li2^v^ and the O7 atom - the Li1^iv^ ion, the O5 atom acts as bidentate bridging to the Li1 and Li2^iii^ ions giving rise to a three-dimensional framework (Fig. 2).

### Experimental

An aqueous solution containing 1 mmol of lithium(I) nitrate and 1 mmol of pyrazine-2,3-dicarboxylic acid dihydrate was boiled with stirring under reflux for 6 h. After cooling to room temperature three drops of 1 N nitric acid were added to maintain pH of 5. Then the solution was left to evaporate to dryness. Deposited single crystal plates were washed with cold ethanol and dried in the air.

### Refinement

The hydrogen atom of carboxylate group was located in a difference map and was refined independently with an isotropic displacement parameter. H atoms bonded to pyrazine ring C atoms were placed in calculated positions with C—H = 0.93 and 0.96 Å and treated as riding on the parent atoms with *U*_iso_(H)= 1.2*U*_eq_(C).

### Crystal data

**Table:**

[Li_2_(C_6_H_3_N_2_O_4_)_2_(NO_3_)]	*F*(000) = 242
*M**_r_* = 241.99	*D*_x_ = 1.783 Mg m^−^^3^
Monoclinic, *P*2_1_	Mo *K*α radiation, λ = 0.71073 Å
Hall symbol: P 2yb	Cell parameters from 2509 reflections
*a* = 4.6273 (1) Å	θ = 3.3–30.7°
*b* = 15.8565 (3) Å	µ = 0.16 mm^−^^1^
*c* = 6.1719 (2) Å	*T* = 293 K
β = 95.598 (2)°	Block, colourless
*V* = 450.69 (2) Å^3^	0.20 × 0.14 × 0.12 mm
*Z* = 2	

### Data collection

**Table:**

Agilent SuperNova (Dual, Cu at zero, Eos) diffractometer	2572 independent reflections
Radiation source: SuperNova (Mo) X-ray Source	2401 reflections with *I* > 2σ(*I*)
Mirror monochromator	*R*_int_ = 0.015
Detector resolution: 16.0131 pixels mm^-1^	θ_max_ = 30.7°, θ_min_ = 3.3°
ω scans	*h* = −5→6
Absorption correction: multi-scan (*CrysAlis PRO*; Agilent, 2011)	*k* = −21→22
*T*_min_ = 0.936, *T*_max_ = 1.000	*l* = −5→8
4032 measured reflections	

### Refinement

**Table:**

Refinement on *F*^2^	Primary atom site location: structure-invariant direct methods
Least-squares matrix: full	Secondary atom site location: difference Fourier map
*R*[*F*^2^ > 2σ(*F*^2^)] = 0.036	Hydrogen site location: inferred from neighbouring sites
*wR*(*F*^2^) = 0.084	H atoms treated by a mixture of independent and constrained refinement
*S* = 1.10	*w* = 1/[σ^2^(*F*_o_^2^) + (0.0337*P*)^2^ + 0.0702*P*] where *P* = (*F*_o_^2^ + 2*F*_c_^2^)/3
2572 reflections	(Δ/σ)_max_ < 0.001
167 parameters	Δρ_max_ = 0.21 e Å^−^^3^
1 restraint	Δρ_min_ = −0.20 e Å^−^^3^

### Special details

**Table:**

Geometry. All e.s.d.'s (except the e.s.d. in the dihedral angle between two l.s. planes) are estimated using the full covariance matrix. The cell e.s.d.'s are taken into account individually in the estimation of e.s.d.'s in distances, angles and torsion angles; correlations between e.s.d.'s in cell parameters are only used when they are defined by crystal symmetry. An approximate (isotropic) treatment of cell e.s.d.'s is used for estimating e.s.d.'s involving l.s. planes.
Refinement. Refinement of *F*^2^ against ALL reflections. The weighted *R*-factor *wR* and goodness of fit *S* are based on *F*^2^, conventional *R*-factors *R* are based on *F*, with *F* set to zero for negative *F*^2^. The threshold expression of *F*^2^ > σ(*F*^2^) is used only for calculating *R*-factors(gt) *etc*. and is not relevant to the choice of reflections for refinement. *R*-factors based on *F*^2^ are statistically about twice as large as those based on *F*, and *R*- factors based on ALL data will be even larger.

### Fractional atomic coordinates and isotropic or equivalent isotropic displacement parameters (Å^2^)

**Table:**

	*x*	*y*	*z*	*U*_iso_*/*U*_eq_	
O1	0.5316 (3)	0.40681 (8)	0.6101 (2)	0.0322 (3)	
O5	0.4586 (3)	0.48672 (8)	0.0630 (2)	0.0297 (3)	
C2	0.4874 (4)	0.27654 (10)	0.4273 (3)	0.0223 (3)	
N4	0.5051 (4)	0.14644 (9)	0.2348 (2)	0.0279 (3)	
N1	0.6499 (3)	0.31421 (9)	0.2871 (2)	0.0278 (3)	
N2	0.1867 (3)	0.47658 (9)	0.0539 (2)	0.0243 (3)	
O2	0.2330 (4)	0.31513 (9)	0.7378 (3)	0.0427 (4)	
C8	0.2425 (4)	0.13507 (11)	0.5462 (3)	0.0261 (3)	
C3	0.4152 (4)	0.19074 (10)	0.4027 (3)	0.0228 (3)	
O7	0.0838 (3)	0.43786 (9)	0.2022 (3)	0.0393 (3)	
O6	0.0354 (3)	0.50501 (10)	−0.1046 (2)	0.0388 (3)	
C5	0.6615 (4)	0.18550 (12)	0.0970 (3)	0.0326 (4)	
H5	0.7223	0.1559	−0.0205	0.039*	
C7	0.4118 (4)	0.33782 (11)	0.6048 (3)	0.0267 (3)	
C6	0.7368 (5)	0.27000 (11)	0.1245 (3)	0.0338 (4)	
H6	0.8500	0.2957	0.0269	0.041*	
Li1	0.7058 (7)	0.44849 (19)	0.3298 (5)	0.0289 (6)	
Li2	0.3903 (7)	0.0133 (2)	0.2244 (5)	0.0300 (6)	
O3	0.2524 (3)	0.05895 (8)	0.5143 (2)	0.0329 (3)	
O4	0.1008 (4)	0.16985 (8)	0.6880 (3)	0.0444 (4)	
H1	0.153 (8)	0.252 (2)	0.713 (6)	0.093 (11)*	

### Atomic displacement parameters (Å^2^)

**Table:**

	*U*^11^	*U*^22^	*U*^33^	*U*^12^	*U*^13^	*U*^23^
O1	0.0444 (8)	0.0222 (6)	0.0311 (7)	−0.0058 (6)	0.0086 (6)	−0.0054 (5)
O5	0.0184 (5)	0.0402 (7)	0.0307 (6)	−0.0033 (5)	0.0045 (4)	0.0052 (5)
C2	0.0242 (7)	0.0194 (7)	0.0236 (7)	0.0013 (6)	0.0044 (6)	0.0004 (6)
N4	0.0352 (8)	0.0224 (7)	0.0275 (8)	−0.0012 (6)	0.0092 (6)	−0.0022 (6)
N1	0.0337 (8)	0.0208 (6)	0.0303 (8)	−0.0019 (6)	0.0103 (6)	0.0006 (6)
N2	0.0223 (6)	0.0230 (6)	0.0285 (7)	−0.0014 (5)	0.0073 (5)	−0.0009 (5)
O2	0.0596 (9)	0.0274 (6)	0.0463 (9)	−0.0092 (7)	0.0320 (7)	−0.0110 (6)
C8	0.0308 (9)	0.0230 (8)	0.0249 (9)	−0.0012 (7)	0.0039 (7)	0.0008 (6)
C3	0.0237 (8)	0.0214 (7)	0.0239 (8)	0.0003 (6)	0.0050 (6)	0.0017 (6)
O7	0.0332 (7)	0.0416 (8)	0.0461 (8)	0.0019 (6)	0.0197 (6)	0.0144 (6)
O6	0.0280 (7)	0.0479 (8)	0.0392 (7)	−0.0007 (6)	−0.0036 (6)	0.0101 (6)
C5	0.0433 (10)	0.0271 (9)	0.0301 (9)	0.0005 (8)	0.0173 (8)	−0.0051 (7)
C7	0.0322 (9)	0.0217 (7)	0.0265 (8)	0.0010 (6)	0.0045 (7)	−0.0022 (6)
C6	0.0426 (11)	0.0258 (8)	0.0356 (10)	−0.0016 (8)	0.0171 (8)	0.0032 (7)
Li1	0.0342 (16)	0.0249 (14)	0.0288 (15)	0.0009 (12)	0.0097 (12)	0.0000 (12)
Li2	0.0382 (16)	0.0234 (13)	0.0287 (15)	0.0015 (13)	0.0052 (13)	0.0008 (12)
O3	0.0495 (8)	0.0207 (6)	0.0293 (6)	−0.0037 (5)	0.0085 (6)	0.0027 (5)
O4	0.0629 (10)	0.0275 (7)	0.0488 (9)	−0.0113 (7)	0.0359 (8)	−0.0059 (6)

### Geometric parameters (Å, º)

**Table:**

O1—C7	1.225 (2)	O2—C7	1.273 (2)
O1—Li2^i^	1.989 (3)	O2—H1	1.07 (4)
Li1—O1	2.086 (3)	C8—O3	1.224 (2)
O5—N2	1.2643 (18)	C8—O4	1.269 (2)
Li1—O5	2.005 (3)	C8—C3	1.530 (2)
Li1—N1	2.158 (3)	O7—Li1^iv^	1.994 (3)
Li1—O7^ii^	1.994 (3)	O6—Li2^v^	2.040 (4)
Li1—O3^i^	1.999 (3)	C5—C6	1.391 (3)
O5—Li2^iii^	2.014 (3)	C5—H5	0.9300
C2—N1	1.341 (2)	C6—H6	0.9300
C2—C3	1.406 (2)	Li1—Li2^i^	3.011 (4)
C2—C7	1.530 (2)	Li2—O1^vi^	1.989 (3)
N4—C5	1.324 (2)	Li2—O5^vii^	2.014 (3)
N4—C3	1.351 (2)	Li2—O6^viii^	2.040 (4)
Li2—N4	2.176 (3)	Li2—O3	2.086 (3)
N1—C6	1.319 (2)	Li2—Li1^vi^	3.011 (4)
N2—O6	1.231 (2)	O3—Li1^vi^	1.999 (3)
N2—O7	1.2359 (19)	O4—H1	1.34 (4)
			
C7—O1—Li2^i^	146.19 (15)	C5—C6—H6	119.5
C7—O1—Li1	118.12 (15)	O7^ii^—Li1—O3^i^	102.51 (15)
Li2^i^—O1—Li1	95.24 (14)	O7^ii^—Li1—O5	98.83 (14)
N2—O5—Li1	118.93 (13)	O3^i^—Li1—O5	98.69 (14)
N2—O5—Li2^iii^	114.63 (14)	O7^ii^—Li1—O1	136.49 (18)
Li1—O5—Li2^iii^	124.59 (14)	O3^i^—Li1—O1	84.57 (13)
N1—C2—C3	120.27 (14)	O5—Li1—O1	122.78 (17)
N1—C2—C7	111.15 (14)	O7^ii^—Li1—N1	88.16 (13)
C3—C2—C7	128.55 (15)	O3^i^—Li1—N1	158.15 (18)
C5—N4—C3	118.59 (14)	O5—Li1—N1	98.41 (14)
C5—N4—Li2	125.60 (15)	O1—Li1—N1	74.76 (11)
C3—N4—Li2	115.76 (14)	O7^ii^—Li1—Li2^i^	127.08 (16)
C6—N1—C2	119.08 (15)	O3^i^—Li1—Li2^i^	43.67 (9)
C6—N1—Li1	125.14 (14)	O5—Li1—Li2^i^	121.60 (15)
C2—N1—Li1	115.41 (13)	O1—Li1—Li2^i^	41.13 (9)
O6—N2—O7	122.70 (15)	N1—Li1—Li2^i^	114.95 (13)
O6—N2—O5	118.37 (14)	O1^vi^—Li2—O5^vii^	102.31 (15)
O7—N2—O5	118.93 (15)	O1^vi^—Li2—O6^viii^	104.59 (16)
C7—O2—H1	114 (2)	O5^vii^—Li2—O6^viii^	94.18 (14)
O3—C8—O4	124.69 (17)	O1^vi^—Li2—O3	84.81 (13)
O3—C8—C3	116.47 (15)	O5^vii^—Li2—O3	171.64 (18)
O4—C8—C3	118.83 (14)	O6^viii^—Li2—O3	88.17 (14)
N4—C3—C2	119.89 (14)	O1^vi^—Li2—N4	141.01 (18)
N4—C3—C8	111.18 (14)	O5^vii^—Li2—N4	97.16 (14)
C2—C3—C8	128.92 (14)	O6^viii^—Li2—N4	107.32 (15)
N2—O7—Li1^iv^	131.81 (15)	O3—Li2—N4	74.49 (12)
N2—O6—Li2^v^	139.98 (15)	O1^vi^—Li2—Li1^vi^	43.63 (9)
N4—C5—C6	121.23 (16)	O5^vii^—Li2—Li1^vi^	145.93 (15)
N4—C5—H5	119.4	O6^viii^—Li2—Li1^vi^	94.92 (13)
C6—C5—H5	119.4	O3—Li2—Li1^vi^	41.41 (9)
O1—C7—O2	123.79 (16)	N4—Li2—Li1^vi^	111.21 (14)
O1—C7—C2	116.83 (15)	C8—O3—Li1^vi^	142.15 (15)
O2—C7—C2	119.38 (15)	C8—O3—Li2	119.94 (14)
N1—C6—C5	120.92 (17)	Li1^vi^—O3—Li2	94.92 (14)
N1—C6—H6	119.5	C8—O4—H1	113.8 (17)
			
C3—C2—N1—C6	1.0 (3)	N2—O5—Li1—Li2^i^	−57.2 (2)
C7—C2—N1—C6	179.39 (16)	Li2^iii^—O5—Li1—Li2^i^	139.21 (19)
C3—C2—N1—Li1	174.35 (15)	C7—O1—Li1—O7^ii^	−88.5 (3)
C7—C2—N1—Li1	−7.3 (2)	Li2^i^—O1—Li1—O7^ii^	97.2 (3)
Li1—O5—N2—O6	175.52 (16)	C7—O1—Li1—O3^i^	168.99 (15)
Li2^iii^—O5—N2—O6	−19.3 (2)	Li2^i^—O1—Li1—O3^i^	−5.28 (15)
Li1—O5—N2—O7	−5.3 (2)	C7—O1—Li1—O5	72.1 (2)
Li2^iii^—O5—N2—O7	159.91 (16)	Li2^i^—O1—Li1—O5	−102.13 (19)
C5—N4—C3—C2	0.3 (3)	C7—O1—Li1—N1	−18.14 (18)
Li2—N4—C3—C2	177.86 (16)	Li2^i^—O1—Li1—N1	167.59 (13)
C5—N4—C3—C8	−179.02 (17)	C7—O1—Li1—Li2^i^	174.3 (2)
Li2—N4—C3—C8	−1.4 (2)	C6—N1—Li1—O7^ii^	−34.8 (2)
N1—C2—C3—N4	−1.3 (3)	C2—N1—Li1—O7^ii^	152.34 (15)
C7—C2—C3—N4	−179.31 (17)	C6—N1—Li1—O3^i^	−155.0 (4)
N1—C2—C3—C8	177.86 (17)	C2—N1—Li1—O3^i^	32.2 (6)
C7—C2—C3—C8	−0.2 (3)	C6—N1—Li1—O5	63.8 (2)
O3—C8—C3—N4	12.0 (2)	C2—N1—Li1—O5	−109.00 (16)
O4—C8—C3—N4	−167.97 (17)	C6—N1—Li1—O1	−174.35 (17)
O3—C8—C3—C2	−167.18 (17)	C2—N1—Li1—O1	12.80 (17)
O4—C8—C3—C2	12.8 (3)	C6—N1—Li1—Li2^i^	−165.38 (18)
O6—N2—O7—Li1^iv^	−32.3 (3)	C2—N1—Li1—Li2^i^	21.8 (2)
O5—N2—O7—Li1^iv^	148.58 (19)	C5—N4—Li2—O1^vi^	112.0 (3)
O7—N2—O6—Li2^v^	1.3 (3)	C3—N4—Li2—O1^vi^	−65.4 (3)
O5—N2—O6—Li2^v^	−179.55 (19)	C5—N4—Li2—O5^vii^	−7.8 (2)
C3—N4—C5—C6	0.9 (3)	C3—N4—Li2—O5^vii^	174.83 (14)
Li2—N4—C5—C6	−176.40 (18)	C5—N4—Li2—O6^viii^	−104.4 (2)
Li2^i^—O1—C7—O2	9.4 (4)	C3—N4—Li2—O6^viii^	78.16 (19)
Li1—O1—C7—O2	−160.34 (19)	C5—N4—Li2—O3	172.54 (18)
Li2^i^—O1—C7—C2	−170.2 (2)	C3—N4—Li2—O3	−4.88 (16)
Li1—O1—C7—C2	20.1 (2)	C5—N4—Li2—Li1^vi^	153.02 (18)
N1—C2—C7—O1	−7.9 (2)	C3—N4—Li2—Li1^vi^	−24.4 (2)
C3—C2—C7—O1	170.27 (18)	O4—C8—O3—Li1^vi^	−43.1 (4)
N1—C2—C7—O2	172.51 (18)	C3—C8—O3—Li1^vi^	136.9 (2)
C3—C2—C7—O2	−9.3 (3)	O4—C8—O3—Li2	162.41 (19)
C2—N1—C6—C5	0.2 (3)	C3—C8—O3—Li2	−17.6 (2)
Li1—N1—C6—C5	−172.44 (19)	O1^vi^—Li2—O3—C8	159.34 (16)
N4—C5—C6—N1	−1.2 (3)	O5^vii^—Li2—O3—C8	10.7 (14)
N2—O5—Li1—O7^ii^	158.55 (14)	O6^viii^—Li2—O3—C8	−95.84 (18)
Li2^iii^—O5—Li1—O7^ii^	−5.1 (2)	N4—Li2—O3—C8	12.71 (19)
N2—O5—Li1—O3^i^	−97.24 (17)	Li1^vi^—Li2—O3—C8	164.6 (2)
Li2^iii^—O5—Li1—O3^i^	99.13 (17)	O1^vi^—Li2—O3—Li1^vi^	−5.27 (15)
N2—O5—Li1—O1	−8.1 (2)	O5^vii^—Li2—O3—Li1^vi^	−153.9 (13)
Li2^iii^—O5—Li1—O1	−171.75 (15)	O6^viii^—Li2—O3—Li1^vi^	99.55 (14)
N2—O5—Li1—N1	69.12 (17)	N4—Li2—O3—Li1^vi^	−151.90 (13)
Li2^iii^—O5—Li1—N1	−94.52 (18)		

Symmetry codes: (i) −*x*+1, *y*+1/2, −*z*+1; (ii) *x*+1, *y*, *z*; (iii) −*x*+1, *y*+1/2, −*z*; (iv) *x*−1, *y*, *z*; (v) −*x*, *y*+1/2, −*z*; (vi) −*x*+1, *y*−1/2, −*z*+1; (vii) −*x*+1, *y*−1/2, −*z*; (viii) −*x*, *y*−1/2, −*z*.

### Hydrogen-bond geometry (Å, º)

**Table:**

*D*—H···*A*	*D*—H	H···*A*	*D*···*A*	*D*—H···*A*
O2—H1···O4	1.07 (4)	1.34 (4)	2.3955 (19)	170 (4)
